# Fgk3 interacts with FgMetR to regulate mycelial growth, conidia development, DON production, and pathogenicity in *Fusarium graminearum*

**DOI:** 10.1007/s44154-026-00342-0

**Published:** 2026-09-14

**Authors:** Min Liu, Ziyun He, Ying Wang, Xuheng Gao, Qing Ma, Wende Liu, Guangfei Tang

**Affiliations:** 1 Anhui Agricultural University School of Life Sciences, National Engineering Laboratory of Crop Stress Resistance Breeding, Changjiang West Road, 130, Shushan District, Hefei, Anhui China; 2 State Key Laboratory for Biology of Plant Diseases and Insect Pests, Institute of Plant Protection, Chinese Academy of Agricultural Sciences, Beijing, 100193 China

**Keywords:** *Fusarium graminearum*, Fgk3, FgMetR, DON, Pathogenicity

## Abstract

Glycogen synthase kinase-3 (Gsk3) is a potential drug target in human medicine, controlling many substrates in cellular processes. In this study, FgMetR was identified as a putative binding partner of Fgk3, the *Fusarium graminearum* ortholog of Gsk3, although the specific functions of both proteins remain uncharacterized. Notably, the physical interaction between FgMetR and Fgk3 was confirmed via yeast two-hybrid (Y2H) assays, and both proteins were shown to co-localize within the nucleus. FgMetR was vital for mycelial growth, conidia development, deoxynivalenol (DON) production, oxidative stress response, and pathogenicity in *F. graminearum*. RNA-seq showed the Fgk3 and FgMetR co-regulated ABC transporters pathway and secondary metabolite biosynthesis. This study identified FgMetR as a new interacting protein of Fgk3, and demonstrated its crucial role in the growth, conidiation, DON production, and virulence of *F. graminearum*. It advanced the understanding of Fgk3 function in filamentous fungi and provided a theoretical basis for preventing Fusarium-related diseases.

**Supplementary Information:**

The online version contains supplementary material available at https://doi.org/10.1007/s44154-026-00342-0.

## Highlights

- FgMetR interacts with Fgk3, and both proteins are co-localized in the nucleus.
- FgMetR is essential for mycelial growth, conidia development, DON production and pathogenicity in* F. graminearum.*
- Fgk3 and FgMetR are involved in the response to oxidative stress and biosynthesis of secondary metabolites, ABC transporters pathway in *F.graminearum.*
- FgMetR represents potential targets for future fungicide development against *F. graminearum.*

**Supplementary Information:**

The online version contains supplementary material available at https://doi.org/10.1007/s44154-026-00342-0.

## Introduction

*Fusarium graminearum* is one of the most destructive plant pathogenic fungi and can infect various grain cereals, including wheat, barley, and oats, as well as maize and rice, reducing crop production and quality of grain by producing deoxynivalenol (DON) mycotoxins (Chen et al. 2019). In addition to being a mycotoxin, DON has also been confirmed to be an important virulence factor of *F. graminearum* and can reduce the defense mechanisms of plants and aggravate Fusarium disease epidemics (Goswami and Kistler 2005; Sella et al. 2014). The application of chemical fungicides remains a cost-effective and efficient approach to mitigate the occurrence of Fusarium diseases and the accumulation of DON contamination (Chen et al. 2024; Li et al. 2019). In China, the fungicides registered for the control of Fusarium diseases are limited to phenamacril, and tebuconazole, which restricts the chemical control strategy for this disease. Moreover, the resistance of Fusarium to these fungicides is increasingly severe, which not only leads to failure of disease control in the fields but also triggers the production of more DON, posing a serious threat to food safety (Tang et al. 2018). Therefore, it is urgent to identify new antifungal targets and novel mechanisms of action of fungicides to manage Fusarium diseases in response to the growing consumer demand for food safety.

Glycogen synthase kinase-3 (Gsk3), a multifunctional serine/threonine kinase, is extensively engaged in regulating diverse cellular processes, such as cell proliferation, differentiation, apoptosis, and metabolism (Liang et al. 2024; Thapa et al. 2023). Given its pivotal role in numerous signaling pathways closely associated with disease development, including the Wnt/β-catenin, PI3K/AKT, and NF-kB signaling pathways, Gsk3 has emerged as a crucial therapeutic target. Consequently, a plethora of Gsk3-targeted inhibitors have been developed and employed in the treatment of human diseases (Eldar-Finkelman and Martinez 2011; Thapa et al. 2023). Clinical investigations have demonstrated the efficacy of Gsk3 inhibitors in a range of cancer types, including pancreatic cancer, ovarian cancer, lung cancer and other malignancies (Zhao et al. 2024). In addition, Gsk3 inhibitors also have certain effects in the treatment of diseases such as diabetes, Alzheimer's disease, and bipolar disorder (Beurel et al. 2015). Among them, lithium (Li) inhibitors, the classic mood stabilizers used to treat bipolar disorder have been the most extensively studied (Klein and Melton 1996). A significant amount of research has shown that Gsk3 is the main target of lithium, and developing new Gsk3 inhibitors presents a promising approach for treating bipolar affective disorder (Chatterjee and Beaulieu 2022). Taken together, Gsk3 exerts a profound impact on the study of human diseases and holds promising research prospects for therapeutic interventions. Nevertheless, investigations into this kinase within the context of fungi have been relatively limited, and its functions in this realm warrant further in-depth exploration.

Gsk3 is a distinctive kinase featuring highly conserved protein structures and phosphorylation properties. However, it plays diverse roles in mediating a wide array of signal pathways across eukaryotic organisms (Cohen and Frame 2001; Tao et al. 2023). A growing body of literature shows that Gsk3 can phosphorylate a variety of targeting proteins, regulate a variety of cellular events, and be phosphorylated by other kinases under different signaling conditions (Wu and Pan 2010). For example, Gsk3-like kinase, GmSK2-8 directly phosphorylates GmNSP1 to mediate salt-inhibited legume–rhizobium symbiosis in soybean (He et al. 2021). In Arabidopsis, a Gsk3-like kinase, BIN2 (brassinosteroid-insensitive 2) kinase is capable of directly phosphorylating the RAPTOR1B (a regulatory-related protein of TOR 1B) to regulate autophagy (Liao et al. 2023). In *Saccharomyces cerevisiae*, four genes, namely MCK1, MDS1 (RIM11), MRK1, and YOL128c, have been reported as homologs of mammalian Gsk3. These findings also disclose that yeast Gsk3 regulates the formation of a complex between the transcription factor Msn2p and DNA, which is crucial for responding to various stresses (Hirata et al. 2003). In *Magnaporthe oryzae*, MoGsk1, a gene functionally homologous to *S. cerevisiae* Gsk3, is functionally characterized to regulate the growth, conidiation and pathogenicity of *M. oryzae* (Zhou et al. 2017). In *Coprinopsis cinerea*, the results show that Gsk3 activity plays an important role in regulating the expression of genes involved in fruiting body initiation and cellular processes (Chan et al. 2024). In *F. graminearum*, one Gsk3 homolog Fgk3 is identified and proven to be required for growth, conidiation, sexual production and virulence (Qin et al. 2015). Recent studies further reveal that Fgk3 regulates chitin synthesis through the carbon catabolite repressor FgCreA in *F. graminearum* (Ni et al. 2024). The multiple functions of Fgk3 suggest that it is a conservative regulator involved in various aspects of fungal development.

In this study, we identify an uncharacterized protein, FgMetR, which interacts with Fgk3. FgMetR is a transcription factor belonging to the basic leucine zipper (bZIP) family. Our results indicate that Fgk3 interacts with FgMetR in the nucleus. Furthermore, the deletion of FgMetR causes a remarkable reduction of mycelial growth, DON production and pathogenicity. RNA-seq analysis found that the Fgk3 and FgMetR co-regulate the expression of downstream genes related to oxidative stress. Moreover, they also regulate the activity of oxidoreductase enzymes, the biosynthesis of secondary metabolites, and the ABC transporters pathway. This finding elucidates the role of Fgk3 in *F.graminearum* and provides novel insights into its regulatory mechanisms in the vegetative growth, stress tolerance, and virulence of pathogenic filamentous fungi.

## Results

### A putative interacting protein FgMetR is identified in Fgk3

**Fig. 1:**
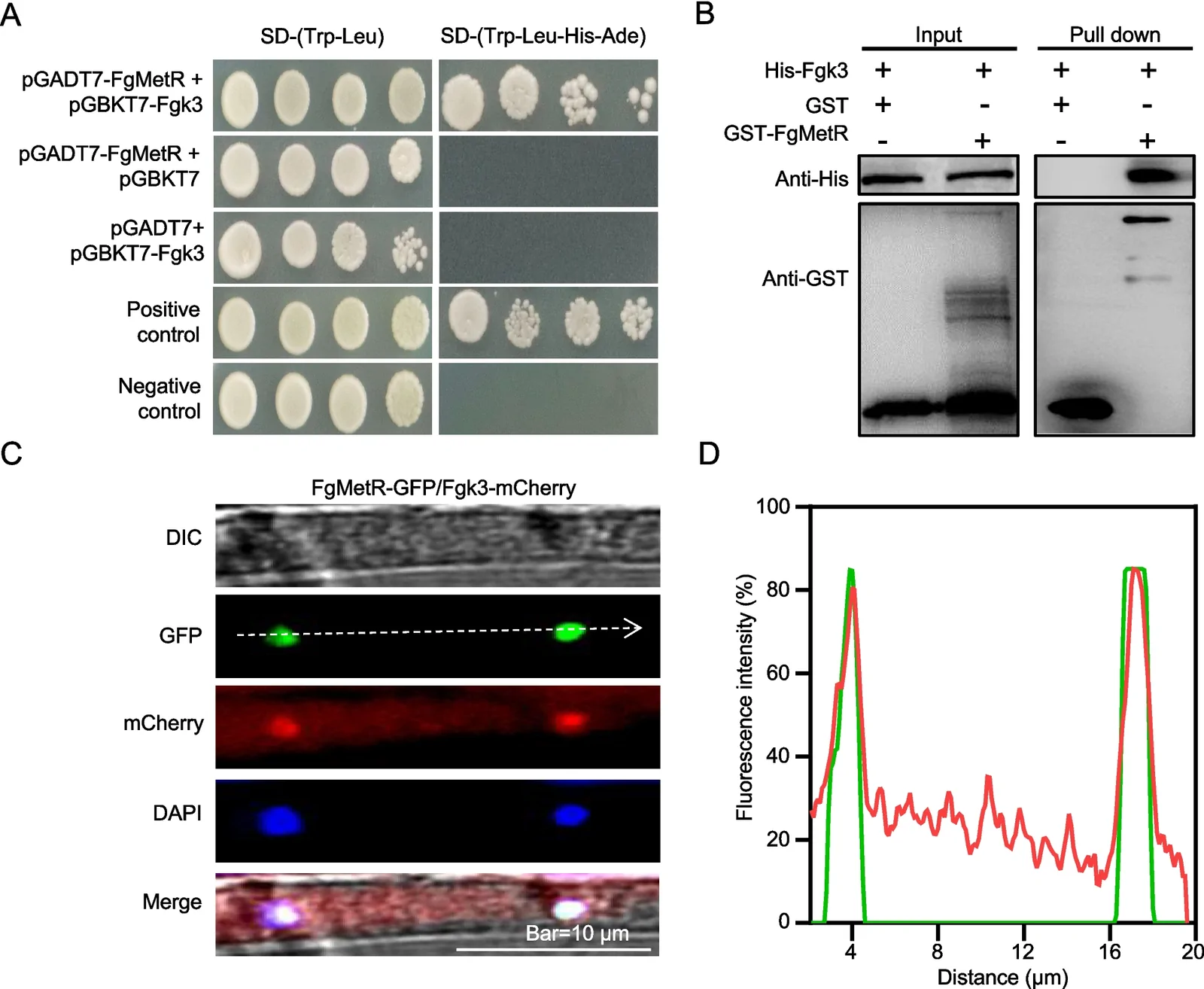
Interaction between FgMetR with glycogen synthase kinase Fgk3. **A** Yeast two-hybrid (Y2H) assay verified the interaction of FgMetR with Fgk3. The pGBKT7-53 and pGADT7-T interactions were positive controls, and the pGBKT7-Lam and pGADT7-T interactions were negative controls. **B** Recombinant GST-FgMetR and GST (negative control) were immobilized on glutathione Sepharose beads and then incubated with His-tagged Fgk3. Following extensive washing to remove unbound proteins, the eluted fractions were subjected to immunoblot analysis. Monoclonal anti-His antibody was used to detect Fgk3, while anti-GST antibody was employed to confirm the integrity of the bait protein. **C** FgMetR-GFP and Fgk3-mCherry were co-transfected into WT for co-localization analysis. The vegetative hyphae of the double-labeled strains were observed under a confocal microscope. DAPI staining was employed to visualize the nuclei, and differential interference contrast imaging was also performed. Scale Bar = 10 µm. **D** ImageJ was used to analyze the fluorescence intensity along the area scanned by the dashed arrow in Fig. 1C. The green line represents FgMetR-GFP, while the red line indicates Fgk3-mCherry. Scale Bar = 10 µm

To investigate the biological function of Fgk3 in *F. graminearum*, we employed a yeast two-hybrid (Y2H) screening strategy using Fgk3 as bait to capture interacting proteins. FgMetR, a transcription factor, was a candidate interacting partner for Fgk3. The physical interaction between Fgk3 and FgMetR was rigorously validated through reciprocal Y2H assays (Fig. 1A), followed by independent confirmation using GST pull-down experiments under in vitro conditions (Fig. 1B). To clarify the spatial relationship between these proteins, we performed subcellular localization studies by co-expressing FgMetR-GFP and Fgk3-mCherry fusion constructs in the wild-type strain. Confocal microscopy analysis of hyphae cultured in yeast extract peptone dextrose (YEPD) medium revealed nuclear co-localization of both proteins, as evidenced by overlapping fluorescence signals with DAPI-stained nuclei (Fig. 1C). Quantitative co-localization analysis using ImageJ software further demonstrated synchronized fluorescence intensity profiles along the hyphal axis (Fig. 1D).

### Identification of *FgMetR* in *F. graminearum*

**Fig. 2:**
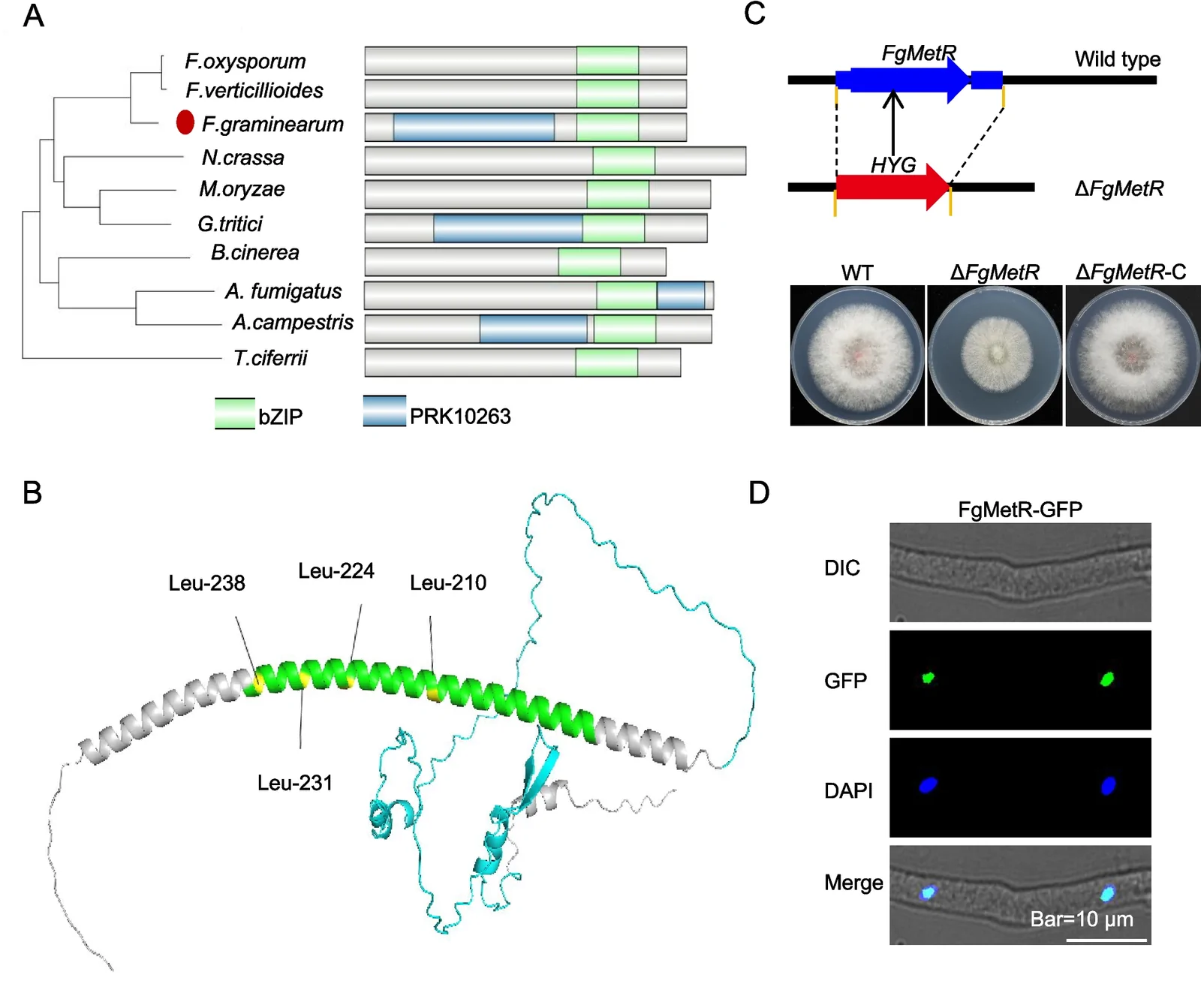
The FgMetR was identified in *F. graminearum.* **A** The phylogenetic tree was constructed with MEGA 11.0 using the neighbour-joining method with 1000 bootstrap replicates. The MetR protein in *F. graminearum* was marked with a red circle (Left panel). The SMART program (http://smart.embl-heidelberg.de) was utilized to predict the conserved domains of MetR, as well as the homologous genes. Blue part represented the PRK10263 domain and green part represented the bZIP domains (Right panel). **B** The protein tertiary structural diagram of FgMetR predicted by AlphaFold3, and labeling the leucine sites in the bZIP domain. **C** Schematic diagram of the principle of hygromycin phosphotransferase (HYG) to replace the target gene (*FgMetR*). The structure of the FgMetR in the wild-type strain and gene replacement events in Δ*FgMetR*. The transcription direction was indicated by the arrow (Top panel). Vegetative growth of the three strains on potato dextrose agar (PDA) at 25 °C for 5 days. (Bottom panel). **D** FgMetR protein was fused with green fluorescent protein (GFP) and transformed into Δ*FgMetR* strain. Images of hyphae were captured using laser scanning confocal microscopy. DAPI staining was used to visualized the nucleus. DIC, differential interference contrast. Scale bars = 10 µm

Bioinformatic analysis of the *F. graminearum* genome through the Ensembl Fungi database (http://fungi.ensembl.org/index.html) revealed a sole bZIP transcription factor, designated *FgMetR* (gene locus FGSG_05171). To identify its orthologs in other fungal species, a BLASTP search was conducted using the amino acid sequence of FgMetR. Phylogenetic reconstruction based on maximum likelihood estimation (MLE) and multiple sequence alignment (Clustal Omega) revealed that FgMetR is highly conserved across these fungal species (Fig. 2A, left panel). Domain analysis showed that the bZIP leucine domain in FgMetR was highly similar among different species (Fig. 2A, right panel). AlphaFold 3 structural prediction (https://alphafoldserver.com/) resolved the tertiary structure of FgMetR, with distinct spatial separation between the N-terminal basic DNA-binding domain (PRK10263 superfamily, blue) and C-terminal leucine zipper dimerization domain (green) (Fig. 2B). To functionally characterize FgMetR, we performed targeted gene replacement of FgMetR in the wild-type strain. The Δ*FgMetR* displayed a remarkable reduction of growth on the PDA medium. The defect in hyphal growth in these mutants was completely restored in the corresponding gene-complementation strains (FgMetR-GFP::Δ*FgMetR*, Δ*FgMetR* − C) under the tested conditions (Fig. 2C). Successful gene replacement and complementation were confirmed by diagnostic PCR and RT-qPCR (Fig. S1A, B). Subcellular localization assays revealed nuclear-specific accumulation of FgMetR-GFP, consistent with its predicted transcriptional regulatory function (Fig. 2D).

### FgMetR regulates conidiation, pathogenicity and DON production of *F. graminearum*

**Fig. 3:**
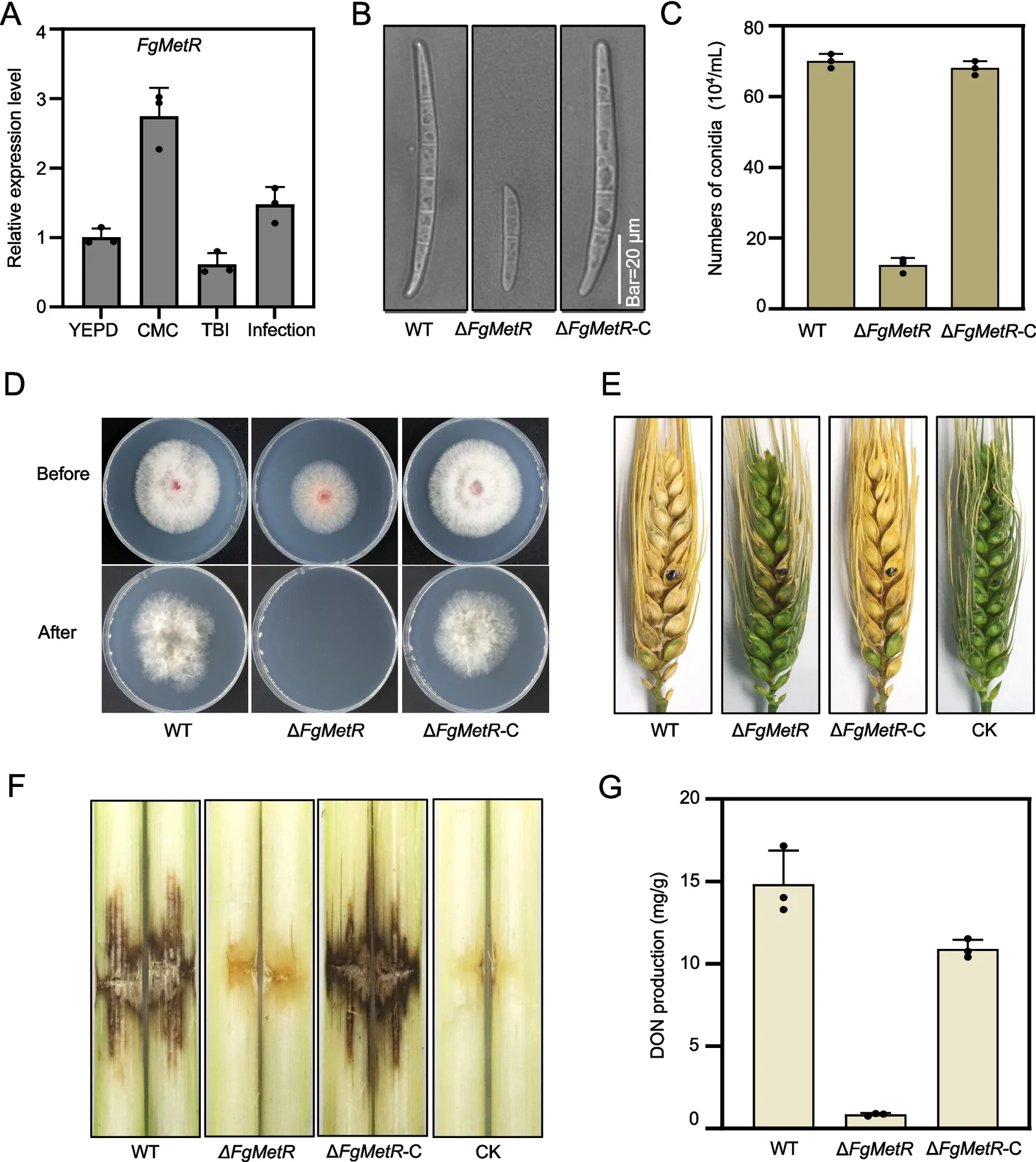
FgMetR plays an important role in conidia development, DON production and pathogenicity of *F. graminearum*. **A** Relative expression levels of FgMetR were cultured in yeast extract peptone dextrose medium (YEPD) (arbitrarily set to1), carboxymethylcellulose sodium medium (CMC), trichothecene biosynthesis induction (TBI) medium and infection conditions. **B** The conidia developmental morphology of the wild-type strain WT, Δ*FgMetR* and Δ*FgMetR*-C in CMC condition for 5 days. Bar = 20 µm. **C** The concentration of conidia of three strains in CMC condition for 5 days. Error bars indicate the standard deviation from three independent experiments. **D** Vegetative growth of the three strains which were grown on the solid PDA medium with cellophane membranes for 3 days at 25 °C (Before part). After the cellophane membrane was removed,vegetative growth of the three strains at 25 °C for another 2 days (After part). **E** Wheat heads inoculated with the three strains were examined for head blight symptoms after 15 days. Black dots mark the inoculated site. **F** Maize stalks inoculated with the three strains were examined for 14 days. **G** DON production of three strains which cultured in trichothecene biosynthesis induction (TBI) medium for 7 days. Error bars indicate the standard deviation from three independent experiments

To gain insight into the biological function of FgMetR, the expression level of *FgMetR* was detected under four critical developmental stages, including hyphal growth (YEPD), conidiation induction (carboxymethyl cellulose, CMC), trichothecene biosynthesis induction (TBI), and during the plant infection stage. RT-qPCR assay showed that high-level *FgMetR* expressions were observed under conidiation condition (Fig. 3A). To further explore its role in conidiation, all strains were cultured in CMC for 5 days. Differences were found in conidial morphology compared to wild-type and Δ*FgMetR*-C. The conidia length of Δ*FgMetR* was shorter than that of the wild-type strain. Moreover, conidia of Δ*FgMetR* contained fewer septum (Fig. 3B). The statistical results showed a significant reduction of about 70% in the number of conidia of the Δ*FgMetR* strain compared to the wild-type and Δ*FgMetR*-C (Fig. 3C). These results indicate that FgMetR is involved in the conidia development of *F. graminearum*.

To assess whether FgMetR played a role in *F. graminearum* virulence, we conducted cellophane penetration assays. As shown in Fig. 3D, Δ*FgMetR* was unable to penetrate the cellophane sheet, indicating a reduction in hyphal infective capacity. Subsequently, we inoculated wheat heads and corn stalks with the WT, Δ*FgMetR*, and Δ*FgMetR*-C strains. The absence of FgMetR led to a significant reduction in pathogenicity (Fig. 3E, F), consistent with the impaired cellophane penetration ability of the mutant (Fig. 3D), suggesting that the virulence defect may be attributed, at least in part, to reduced hyphal infective capacity. DON is an important virulence factor. We used the DON detection kit to determine the DON content of WT, Δ*FgMetR*, and Δ*FgMetR*-C. The results revealed a 14-fold reduction in DON production in Δ*FgMetR* compared to the WT and Δ*FgMetR*-C (Fig. 3G). Collectively, these results highlight the crucial role of FgMetR in conidiation, pathogenicity, and DON production in *F. graminearum*.

### FgMetR responses to various stresses

**Fig. 4:**
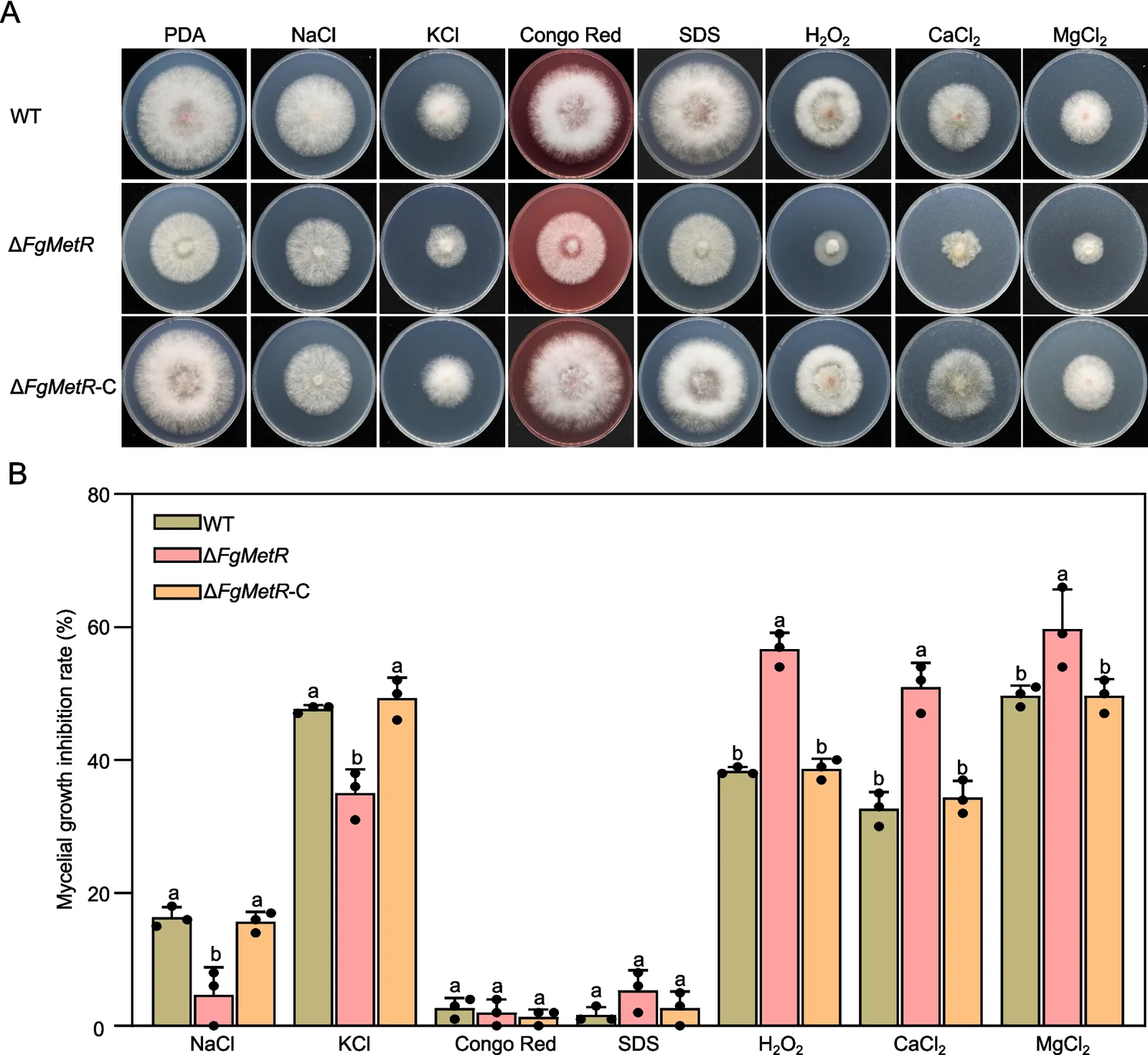
FgMetR is involved in the stress response of *F. graminearum*. **A** Colony morphology of WT, Δ*FgMetR* and Δ*FgMetR*-C strains grew on PDA plates supplemented with NaCl (0.7 M), KCl (1 M), Congo Red (0.2 g/L), SDS (10%), H_2_O_2_ (0.01 M), CaCl_2_ (0.3 M) or MgCl_2_ (0.5 M) at 25 °C for 5 days. **B** Growth inhibition rates of all tested strains were measured after each strain was incubated on PDA supplemented with each stress compound for 5 days. Error bars indicate the standard deviation from three independent experiments. The letters “a” and “b”, in the bars represent significant differences according to the LSD test at *P* = 0.05

To determine whether FgMetR responds to environmental stress, we conducted stress experiments with osmotic stress (NaCl, KCl), cell wall stress (SDS, Congo Red), oxidative stress (H_2_O_2_), and metal ion stress (CaCl_2_, MgCl_2_) (Fig. 4A). The statistical results showed that Δ*FgMetR* did not respond to cell wall stress compared to wild type, but showed higher resistance to osmotic stress and higher sensitivity to oxidative stress and metal ion stress (Fig. 4B). In summary, these results suggest that FgMetR is necessary to modulate the osmotic, oxidative and metal ionization responses of *F. graminearum* to abiotic stress.

### Fgk3 and FgMetR co-regulate the gene transcription

**Fig. 5:**
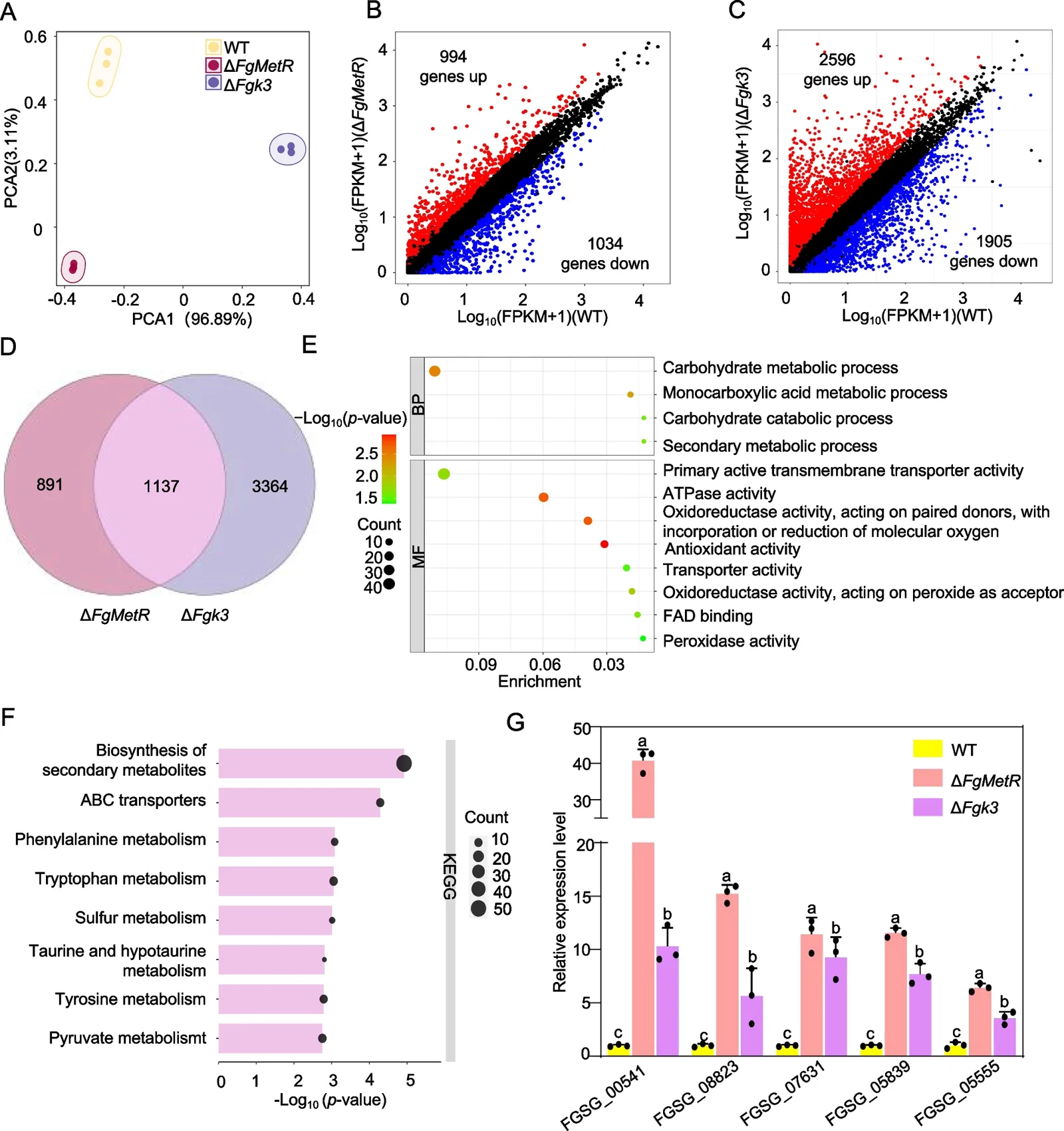
Transcriptome profiles regulated by Fgk3 and FgMetR in *F. graminearum*. **A** Principal component analysis was used to assess inter-group differences and sample duplication within groups. **B** The volcano statistics of up- and down- regulated DEGs in Δ*FgMetR*. **C** The volcano statistics of up- and down- regulated DEGs in Δ*Fgk3*. **D** Venn diagram showing the number of DEGs andco-regulated genes in Δ*FgMetR* and Δ*Fgk3* strains. **E** Gene Ontology (GO) enrichment analysis of the co-regulated genes in Fgk3 and FgMetR. **F** Kyoto Encyclopedia of Genes and Genomes (KEGG) enrichment analysis of the co-regulated genes in Fgk3 and FgMetR. **G** Expression analyses of genes related to ABC transporter activity. The letters “a” and “b”, in the bars represent significant differences according to the LSD test at *P* = 0.05

To facilitate comparison with Fgk3, we assessed the growth phenotype of the Δ*Fgk3* mutant on PDA medium. Consistent with previous reports (Qin et al. 2015), the Δ*Fgk3* mutant exhibited a significant reduction in vegetative growth compared to the wild-type strain (Fig. S2). To further investigate the roles of the Fgk3 and FgMetR, we conducted RNA-seq analysis of WT, Δ*FgMetR* and Δ*Fgk3*. Principal component analysis revealed significant differences among WT, Δ*FgMetR*, and Δ*Fgk3*, with good repeatability observed among the three replicates within each group (Fig. 5A). The volcano plot demonstrated that 2028 and 4501 differentially expressed genes (DEGs) were identified in Δ*FgMetR* and Δ*Fgk3* strains, respectively (Table S2). Among the DEGs identified by Δ*FgMetR*, 994 genes were up-regulated and 1034 genes were down-regulated (Fig. 5B). 2596 genes were up-regulated and 1905 genes were down-regulated in Δ*Fgk3* (Fig. 5C). Venn diagram showed a significant overlap in DEGs between the Δ*FgMetR* and Δ*Fgk3* strains (1137 genes, representing ∼67% of the DEGs in Δ*FgMetR*) (Fig. 5D). To further understand the functions of these genes, we performed gene function enrichment analysis. Under the confidence threshold of *p*-value < 0.05, Gene Ontology (GO) enrichment analysis indicated that 35 genes were involved in the carbohydrate metabolic process, and 23 genes co-regulated by Δ*FgMetR* and Δ*Fgk3* participated in oxidoreductase activity in response to oxidative stress, which was consistent with the results of our previous oxidative stress experiments (Fig. 5E). The Kyoto Encyclopedia of Genes and Genomes (KEGG) KEGG enrichment analysis of co-regulated genes showed that genes were enriched in the biosynthetic pathway of secondary metabolites,ABC transporters pathway, and oxidative stress (Fig. 5F). To investigate the molecular basis of the stress-related phenotypes of Δ*FgMetR*, we performed targeted KEGG enrichment analysis of DEGs. Pathways significantly enriched included those involved in oxidative stress (Glutathione metabolism, *p* = 0.0024; Taurine and hypotaurine metabolism, *p* = 0.0114), osmotic regulation (Glycerolipid metabolism, *p* = 0.0149), and ion homeostasis (Nitrogen metabolism, *p* = 0.0229) (Table S3). The reliability of the RNA-seq data was further validated by RT-qPCR using five representative genes (Fig. 5G). In summary, Fgk3 and FgMetR co-regulate these key biological processes such as the secondary metabolisms, ABC transporters, and oxidative stress.

### Proposed model of Fgk3 and FgMetR functions in *F. graminearum*

**Fig. 6:**
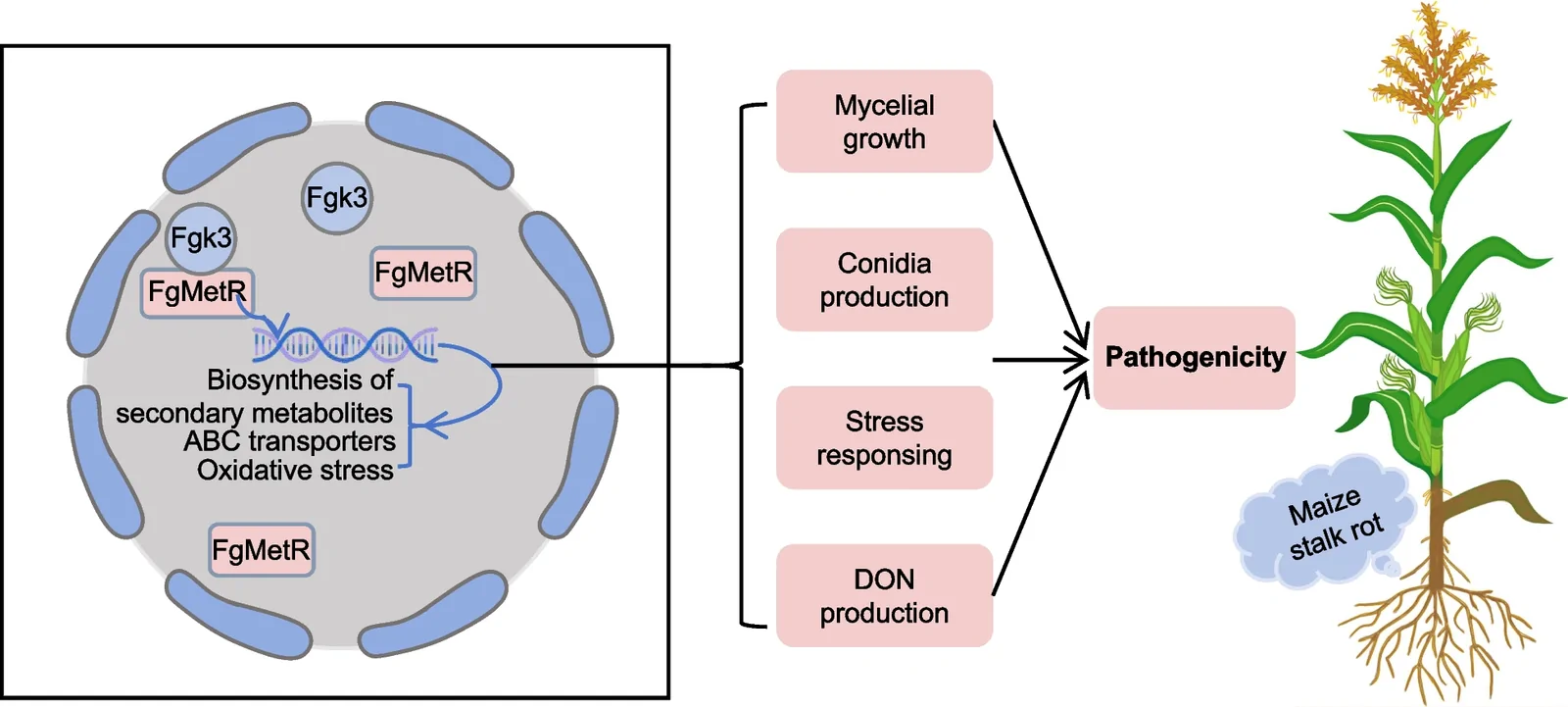
Proposed model of Fgk3 and FgMetR functions in *F. graminearum*. Glycogen synthase kinase Fgk3 interacts with transcription factor FgMetR in the nucleus, and the two proteins co-regulate downstream gene expression. The kinase Fgk3 may phosphorylate the transcription factor FgMetR, thus regulating the expression of biosynthesis of secondary metabolites, ABC transporters and Oxidative-related genes.Thereby exerting a significant impact on the growth, conidiation, stress responsing, DON production and ultimately influencing the pathogenicity of *F. graminearum*

Fgk3, a glycogen synthase kinase in *F. graminearum*, serves as a potential target for fungicides. In this study, we identified a novel interacting protein of Fgk3, namely FgMetR. FgMetR is a transcription factor. We found that it regulates mycelial growth, impacts conidia development, reduces mycelial penetration ability, and decreases the synthesis of DON. Ultimately, we verify that FgMetR influences the pathogenicity of *F. graminearum*. Meanwhile, transcriptome data analysis indicates that the Fgk3 and FgMetR jointly regulate the secondary metabolic pathway, the ABC transport pathway, and the response to oxidative stress. The interaction between Fgk3 and FgMetR provides a foundation for further exploration of their potential as antifungal targets in *F. graminearum* (Fig. 6).

## Discussion

Gsk3 was a multifunctional protein kinase and played a role in a wide range of cellular processes, including differentiation, growth, motility, and apoptosis (Forde and Dale 2007). In mammals, Gsk3 was a validated target for the treatment of diseases (Osolodkin et al. 2011). In humans, over 100 substrates and interacting molecules have been proposed to be associated with Gsk3 (Wang et al. 2022). In plants, Gsk3-like kinases were involved not only in a wide variety of plant growth and developmental processes but also in various plant stress responses (Mao et al. 2021). Nevertheless, the number of identified substrates in plants remained limited. Abundant studies had demonstrated that Gsk3-like kinases performed diverse functions by phosphorylating different substrates (Youn and Kim 2015). Gsk3 orthologs were highly conserved and had been identified in some plant-pathogenic fungi (Grütter et al. 2012). Previous research had shown that Fgk3, a Gsk3 homolog, played a crucial role in the growth, production of DON, pathogenicity, and stress responses within the filamentous ascomycete *F.graminearum* (Qin et al. 2015). These results indicated that Fgk3 played a role in several biological pathways in Fusarium and could serve as a target protein for developing inhibitors aimed at Fusarium. However, the biological pathways involved in regulating Fgk3 and its substrates or related interacting proteins were rarely reported. In the present study, we screened for the reciprocal protein FgMetR of Fgk3 in a yeast library and verified the physical interaction using yeast two-hybrid and pull-down (Fig. 1A, B). FgMetR was co-localized with Fgk3 in the nucleus (Fig. 1C). Since Fgk3 was a kinase, this co-localization implied that Fgk3 may regulate the activity of FgMetR through phosphorylation modifications. Nevertheless, this article does not include research on phosphorylation modifications. Our data indicate that Fgk3 and FgMetR physically interact, share substantial transcriptional targets, and exhibit similar mutant phenotypes, suggesting their involvement in a common regulatory network. However, this overlap is equally compatible with FgMetR acting as a direct downstream effector of Fgk3 or with the two regulators functioning through independent parallel pathways that converge on shared target genes; future epistasis analyses, such as double-mutant phenotyping, will be required to distinguish these possibilities. Furthermore, given that Fgk3 is a conserved protein kinase and both proteins co-localize in the nucleus, it is plausible that Fgk3 modulates FgMetR activity via phosphorylation, although this hypothesis awaits direct kinase assays or phosphoproteomic validation. Similarly, whether the nuclear localization of FgMetR depends on Fgk3 remains unknown and warrants examination in the Δ*Fgk3* background. Collectively, our current conclusions are confined to the observed physical interaction and transcriptional co-regulation; mechanistic inferences regarding direct phosphorylation, regulation of subcellular localization, or epistatic relationships require systematic validation through future overexpression analyses, double-mutant construction, localization assays, and phosphorylation studies.

The basic region/leucine zipper motif (bZIP) transcription factors were proteins that consisted of a basic region responsible for mediating sequence-specific DNA binding and a leucine zipper region necessary for dimerization. This class of transcription factors was among the most widespread and conserved in eukaryotes (Jakoby et al. 2002). In recent years, members of bZIPs had been identified in several fungi. For example, 9 members of the bZIP family were present in *Neurospora crassa* (Tian et al. 2011), 15 in *Aspergillus flavus* (Zhao et al. 2022), 22 in *Magnaporthe oryzae* (Tang et al. 2015) and 22 in *F. graminearum* (Son et al. 2011), 26 bZIP genes in *Coniothyrium chrysosperma* (Wen et al. 2021), 34 in *Coniothyrium minitans* (Xu et al. 2020), 28 in *Ustilaginoidea virens* (Yin et al. 2017) and 38 in *Phytophthora infestans* (Gamboa-Meléndez et al. 2013). In this study, a bZIP family transcription factor FgMetR was identified in *F. graminearum* (Fig. 2). The biological phenotypic results demonstrated that FgMetR was important for growth, conidiation, DON production, virulence and stress response (Figs. 3 and 4), which was consistent with previous reports (Park et al. 2024). MetR, a member of the bZIP family of transcription factors, had been the subject of extensive research. AaMetR was sensitive to H_2_O_2_ and many reactive oxygen species (ROS)-generating compounds, indicating that AaMetR was essential for oxidative tolerance in *Alternaria alternata* (Gai et al. 2022, 2019). In *Serratia marcescens*, a Gram-negative bacterium, MetR was also found to be related to tolerance to H_2_O_2_ (Pan et al. 2020). Compared with the results of our oxidative stress experiments, it was found that Δ*FgMetR* exhibited functional properties that were more sensitive to H_2_O_2_ in *F. graminearum*, which may be one of the reasons for the reduced pathogenicity of Δ*FgMetR*.

Interactions between kinases and transcription factors regulated the expression of associated genes, a crucial mechanism for cells to respond to external stimuli. Hog1 interacted with the transcription factor Sko1 and regulated its transcriptional activity by phosphorylating Sko1, thereby affecting the osmotic pressure adaptation of cells (Proft and Struhl 2002). To clarify the biological significance of the interaction between FgMetR and Fgk3, this study analyzed the transcriptome data of the two gene deletion mutants. The results showed that Fgk3 and FgMetR co-regulated the expression of more than 70 redox-related genes. GO analysis revealed the crucial involvement of genes co-regulated by Fgk3 and FgMetR in oxidative stress-related biological processes, with a particular emphasis on those implicated in peroxidase activity. Alterations in the expression of these genes were instrumental in facilitating cellular maintenance of normal physiological functions under oxidative stress conditions, thereby contributing to the mitigation of oxidative damage (Fig. 5). The functional study of the Fgk3 gene revealed that the Δ*Fgk3* mutant exhibited sensitivity to H_2_O_2_ stress, which was consistent with the results observed for the Δ*FgMetR* mutant in this study (Fig. 4). Therefore, we hypothesized that Fgk3 may influence the activity of FgMetR through interaction, thereby regulating the expression of antioxidant-related genes and ultimately affecting the response of *F. graminearum* to H_2_O_2_ oxidative stress. Oxidative stress in filamentous fungi was frequently associated with the synthesis of secondary metabolites. The glutathione and thioredoxin reductase systems represent significant mechanisms through which filamentous fungi can respond to exogenous oxidative stress. The deletion of the glutathione reductase gene *glrA* in *Acremonium chrysogenum* resulted in an increased sensitivity of mycelia to hydrogen peroxide, with the synthesis of cephalosporin being completely blocked (Nguyen et al. 2025). A KEGG analysis of the genes co-regulated by Fgk3 and FgMetR revealed a significant enrichment of genes associated with secondary metabolite synthesis and ABC transporters, which was in alignment with previous results. The significant decrease in DON production of FgMetR knockout mutants strongly supported our hypothesis. Moreover, DON had been determined not only to be a secondary metabolite but also a pathogenic factor, which played an important role in the process of Fusarium infection (Desmond et al. 2008; Luo et al. 2023). Consequently, there was sufficient evidence to suggest that the interaction between Fgk3 and FgMetR primarily regulated the response of Fusarium to oxidative stress and affected the synthesis of secondary metabolites such as DON through oxidoreductase-related genes, which ultimately affected the pathogenicity of Fusarium. The extensive utilization of bioinformatics technology enabled the virtual screening of key proteins targeting pathogenic fungi, thereby significantly reducing the time and economic cost of fungicide development with new mechanisms of action. In this study, the interaction between FgMetR and Fgk3 was investigated to elucidate its role in the pathogenic process of Fusarium. These findings suggested that FgMetR and Fgk3 could serve as potential targets for Fusarium inhibitors, paving the way for the development of novel chemical structures and mechanisms of action of fungicides. Moreover, to combat the resistance, residue, and resurgence of chemical fungicides, the employment of RNA interference technology facilitates the development of broad-spectrum fungicides that are capable of selectively targeting other plant pathogenic fungi based on the conservation of Gsk3 and MetR sequences. This study provides mechanistic insights into the function of Gsk3 in filamentous fungi and offers a foundation for further exploration of Fgk3 and FgMetR as potential antifungal targets in *F. graminearum*.

## Conclusion

In this study, a putative interacting protein of glycogen synthase kinase Fgk3, FgMetR, has been identified. FgMetR was characterized as having an impact on mycelial growth, conidia production, mycelial penetration ability, and the biosynthesis of deoxynivalenol (DON), thereby exerting a significant influence on the pathogenicity of *Fusarium graminearum*. The Fgk3 and FgMetR co-regulate ABC transporters, oxidoreductase activity, and the biosynthesis of secondary metabolites.This study reveals the regulatory role of Fgk3 and FgMetR in *F. graminearum.* It provides a theoretical foundation for the development of new fungicides targeting the Fgk3 interaction network.

## Materials and methods

### Strains and culture conditions

*Fusarium graminearum* standard strain (WT) was used in this study. WT, the Δ*FgMetR* and Δ*FgMetR* − C strains were cultured on potato dextrose agar (PDA) medium at 25 °C in the dark. The carboxymethylcellulose sodium medium (CMC) was employed to cultivate the conidia of *F. graminearum*. Mycelial samples were prepared using yeast extract peptone dextrose medium (YEPD) (Tang et al. 2020). Conversely, the trichothecene biosynthesis induction (TBI) medium was used to culture *F. graminearum* for the production of deoxynivalenol (DON) toxins (Menke et al. 2012).

### Constructed the deleted strains and complemented strains

The sequence of the target gene *FgMetR* (FGSG_05171) was retrieved and downloaded from the fungal gene sequence database (http://fungidb.org/fungidb/). The double-joint PCR was used to construct the knockout cassette, and the cassette was transformed into protoplasts of the *F. graminearum* (WT) using the methodology described by the previous report (Yu et al. 2014). Positive colonies were selected by hygromycin (*HYG*) resistance. The Δ*FgMetR* and Δ*FgMetR* − C were verified by PCR (Fig. S1A) and RT-qPCR with specific primers (Fig. S1B). The open reading frame (ORF) regions of the target gene and its promoter region were amplified by PCR to construct the complemented strain. Transformants were selected for their resistance to geneticin (G418). Fluorescence microscopy was utilized to determine whether green fluorescence was generated in the complemented strain. All specific primers are presented in Table S1.

### Constructed the phylogenetic tree and related protein domains

To identify the protein structure and orthologs of the *MetR* gene of *F. graminearum*, the protein domain of MetR was found in NCBI. The search identified the FGSG_05171 locus, a 1585-bp gene encoding a 281-amino-acid protein, which was designated as FgMetR. The BLASTP result predicted that FgMetR had a bZIP domain and a PRK10263 superfamily domain. The predicted conserved domains in the protein were identified using the Illustrator for Biological Sequences (https://ibs.renlab.org/#/server). To gain a deeper understanding of the protein conformation of the gene, the tertiary structure of the protein was visualized by AlphaFold3 software (https://alphafoldserver.com/). An in-depth analysis was performed by Pymol software (Jumper et al. 2021), where the leucine sites in the leucine zipper domain (bZIP) were annotated. The MetR amino acid sequences of various fungi were retrieved from NCBI and aligned using the ClustalW algorithm. A phylogenetic tree was constructed based on the amino acid sequences of all species using MEGA 11.0 with the neighbor-joining method. Bootstrap values from 1000 replications are indicated on the branches (Yan and Shim 2020). The domains of all homologous Proteins were simply visualized with IBS.

### Yeast two-hybrid experiment (Y2H)

To construct plasmids for yeast two-hybrid analyses, the coding sequence of each tested gene was amplified from the cDNA of WT. The open reading frames (ORFs) of the FgMetR and Fgk3 were inserted into the pGBKT7 or pGADT7 vector (Stellberger et al. 2012). The plasmids were co-transformed into the *S. cerevisiae* strain AH109 protoplasts (Yu et al. 2014). Transformants were initially cultured on basal medium (SD) lacking leucine (Leu) and tryptophan (Trp) (SD-L-T) at 28 °C for 3 days. Subsequently, they were transferred to basal medium (SD) lacking leucine (Leu), tryptophan (Trp), histidine (His), and adenine (Ade) (SD-L-T-H-A) for 4 days to assess the interaction. Additionally, the plasmids pGBKT7-53 and pGADT7-T served as positive controls, while the plasmids pGBKT7-Lam and pGADT7-T served as negative controls. Three independent experiments were performed to confirm the results.

### In vitro GST pull-down assay

In the in vitro GST pull-down assay, the plasmids GST-FgMetR and His-Fgk3 were separately transformed into the *E. coli* strain BL21. The soluble proteins were then incubated with 30 μL of glutathione agarose beads (Invitrogen) at 4 °C for 4 h. After incubation, the beads were washed three times, and the presence of His-Fgk3 was detected by immunoblotting (IB) with an anti-His antibody.

### Cellophane membrane penetration assay

For the penetration assays, cellophane membranes were autoclaved at 121 °C for 20 min. Sterile cellophane membranes were then placed on the surface of the solid potato dextrose agar (PDA) medium. Subsequently, each strain was inoculated on top of the cellophane membrane and cultured for 3 days. After removing the cellophane membrane, the newly-formed colonies were allowed to grow on the PDA medium for an additional 2 days. The penetration assay was repeated three times (Huang et al. 2024).

### Determination of DON mycotoxin

The DON ELISA Kit from Shanghai Youlong Biotechnology Company was employed to measure the DON content. The test strains were incubated in the trichothecene biosynthesis induction (TBI) medium at 25 °C on a shaker at 160 rpm for 7 days. Three independent experiments were carried out to validate the results.

### Pathogenicity assay

To assess the virulence of maize stalks (B73) and Wheat heads, mycelia were inserted into the middle of wheat heads with toothpicks before the grain filling stage and then grown under natural conditions for 15 days. The conidia concentration was adjusted to 1 × 10^6^/mL, and the floating conidia solution was injected into live maize stalks grown for 8 weeks and naturally grown for 14 days. At least ten wheat heads or maize stalks are inoculated for each strain (Feng et al. 2018).

### RNA-seq sequencing and analysis

The WT, Δ*FgMetR* strains, and Δ*Fgk3* strains were cultured in yeast extract peptone dextrose (YEPD) medium for 24 h. Then, the mycelia were collected and sent to Novogene (Novogene Co., Ltd, Tianjin Biotech, Beijing, China) for RNA-seq analysis. Each strain was replicated three times. We used the Novomagic Platform for data analysis (https://magic.novogene.com/customer/main#/homeNew).

### Statistical analysis

The physiological and biochemical data were processed using the Data Processing System (DPS) 7.05 software (Hangzhou Ruifeng Information Technology Co., Ltd., Hangzhou, China). For the means of multiple groups, two-way analysis of variance (ANOVA) combined with Tukey’s multiple comparison test was employed to assess significant differences, and a significance threshold of *p* < 0.05 was set.

## Supplementary Information

Supplementary Material 1: Fig. S1. PCR, RT-PCR validation of Δ*FgMetR*. A PCR validation of Δ*FgMetR* and Δ*FgMetR*-C. B Semiquantitative RT-PCR validation of the expression level of FgMetR in WT, Δ*FgMetR* and Δ*FgMetR*-C. *Actin* served as the control.

Supplementary Material 2: Fig. S2. Colony morphology of WT, Δ*Fgk3*, and Δ*Fgk3*-C strains. Vegetative growth of the WT, Δ*Fgk3*, and Δ*Fgk3*-C on potato dextrose agar (PDA) at 25 °C for 5 days.

Supplementary Material 3.

Supplementary Material 4.

Supplementary Material 5.

## Data Availability

All data that support the findings of this study are available within the manuscript and the Supporting Information. The raw RNA-seq data (accession no. PRJNA1211511) have been deposited in the National Center for Biotechnology Information (NCBI) Sequence Read Archive (SRA) (https://www.ncbi.nlm.nih.gov/sra/?term=PRJNA1211511).

## Abbreviations

DON: Deoxynivalenol
Gsk3: Glycogen synthase kinase-3
*F. graminearum*: *Fusarium graminearum*
Li: Lithium
RAPTOR1B: A regulatory-related protein of TOR 1B
PDA: Potato dextrose agar
CMC: Carboxymethylcellulose sodium medium
YEPD: Yeast extract peptone dextrose medium
TBI: Trichothecene biosynthesis induction
WT: Fusarium graminearum
HYG: Hygromycin
PCR: Polymerase chain reaction
RT-qPCR: Real-Time qPCR
ORF: Open reading frame
G418: Geneticin
NCBI: National Center for Biotechnology Information
SD: Basal medium
Leu: Leucine
Trp: Tryptophan
His: Histidine
Ade: Adenine
IB: Immunoblotting
DPS: Data processing system
Y2H: Yeast two-hybrid
NaCl: Sodium chloride
KCl: Potassium chloride
SDS: Sodium dodecyl sulfate
H_2_O_2_: Hydrogen peroxide
CaCl_2_: Calcium chloride
DEGs: Differentially expressed genes
GO: Gene Ontology
KEGG: Kyoto encyclopedia of genes and genomes
MgCl_2_: Magnesium chloride
