# Supplementary material for: Fgk3 interacts with FgMetR to regulate mycelial growth, conidia development, DON production, and pathogenicity in Fusarium graminearum
Source: Stress Biol. 2026 Sep 14;6(1):64. doi: 10.1007/s44154-026-00342-0 (PMC13574754; doi:10.1007/s44154-026-00342-0)
Supplement: Supplementary file 1 [file 44154_2026_342_MOESM1_ESM.pdf]

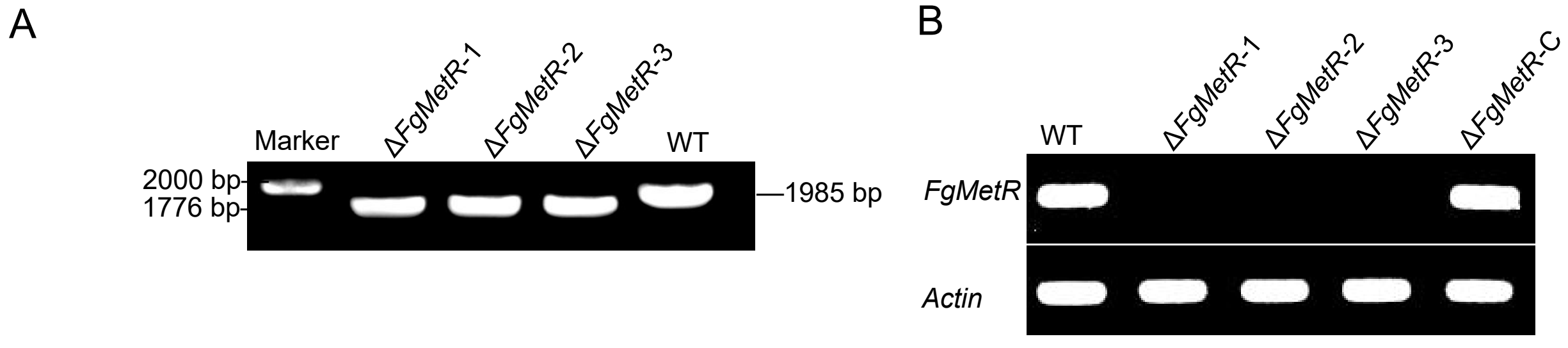

Fig. S1 PCR， RT-PCR validation of Δ*FgMetR*.

A PCR validation of Δ*FgMetR* and Δ*FgMetR*-C. **B** Semiquantitative RT-PCR validation of the expression level of FgMetR in WT, Δ*FgMetR* and Δ*FgMetR*-C. *Actin* served as the control.
