# Supplementary material for: Fgk3 interacts with FgMetR to regulate mycelial growth, conidia development, DON production, and pathogenicity in Fusarium graminearum
Source: Stress Biol. 2026 Sep 14;6(1):64. doi: 10.1007/s44154-026-00342-0 (PMC13574754; doi:10.1007/s44154-026-00342-0)
Supplement: Supplementary file 2 [file 44154_2026_342_MOESM2_ESM.pdf]

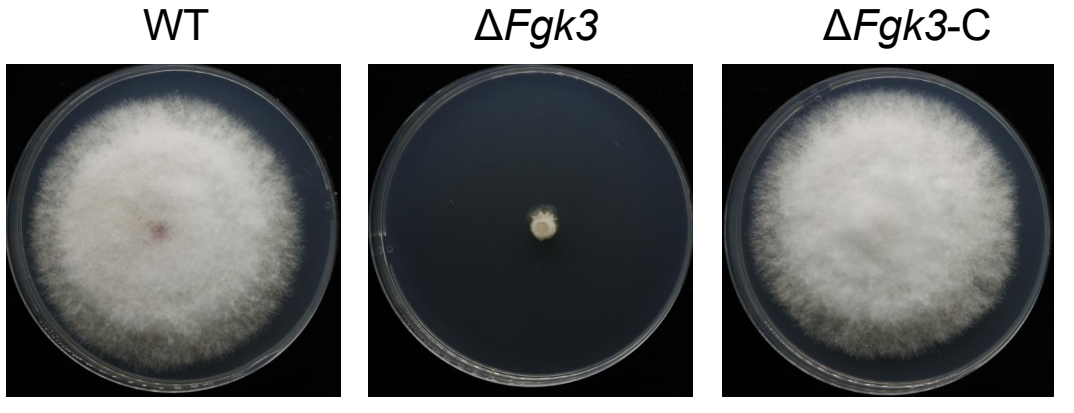

Fig. S2 Colony morphology of WT, Δ*Fgk3*, and Δ*Fgk3*-C strains.
Vegetative growth of the WT, Δ*Fgk3*, and Δ*Fgk3*-C on potato dextrose agar (PDA) at 25°C for 5 days.
